# Comparing masking and habituation roles in saccadic omission of stimuli optimized for intra-saccadic vision

**DOI:** 10.1167/jov.26.7.5

**Published:** 2026-07-07

**Authors:** Umberto Calleri, Antonella Pomè, Eckart Zimmermann

**Affiliations:** 1 Institute for Experimental Psychology, Heinrich Heine University Düsseldorf, Düsseldorf, Germany

**Keywords:** backward masking, saccadic omission, habituation, motion perception

## Abstract

Saccadic eye movements produce a rapid visual motion stimulation that remains unseen, a phenomenon known as saccadic omission. We have recently suggested that the sensorimotor system selectively habituates to, and thereby omits, the visual contingencies of saccades. In previous studies, a passive mechanism of saccadic omission was proposed in which the post-saccadic image serves as a backward mask to hide intra-saccadic motion from awareness. Here, we directly compared these theories using stimuli optimized for intra-saccadic vision: high-speed gratings that become visible only during the execution of saccades. In Experiment 1, we tested backward masking for three intra-saccadic motion stimuli (sudden-onset stationary grating, sudden-onset moving grating, and smooth-onset moving grating) by presenting a post-saccadic mask at saccade offset. Masking strongly reduced subjective motion report for sudden-onset stimuli but had little effect on smooth-onset motion, suggesting that gradual trans-saccadic transitions can bypass visual masking. In Experiment 2, repeated exposure reduced the detection of smooth-onset motion, but sudden-onset motion showed no reliable change. Thus, intra-saccadic habituation acts as an adaptive reduction in sensitivity to predictable motion associated with eye movements. Together, the results reveal complementary roles of masking and habituation in maintaining visual stability: Transient masking primarily impacts sudden intra-saccadic transients, whereas habituation tunes perception to predictable self-generated motion.

## Introduction

Our visual perception evolved to detect motion in the environment. However, moving our eyes produces motion on the retina that, in principle, could be interpreted as environmental motion unless they are appropriately compensated (Sperry, 1950). In practice, observers are typically unaware of the self-produced retinal stimulation, as reflected in the difficulty of seeing one's eye movements in a mirror (Campbell & Wurtz, 1978). Researchers have sought to explain the absence of motion perception during saccades in terms of either active or passive mechanisms (for review, see Wurtz, 2018). In an active saccadic omission mechanism, a copy of the motor command (i.e., an efference copy) informs visual areas about an upcoming eye movement (Sperry, 1950; von Holst & Mittelstaedt, 1950). In consequence, processing of the self-produced visual motion will be treated differently by these areas (Sommer & Wurtz, 2008). In a passive saccadic omission mechanism, no such signal is necessary; instead, properties of the sensory input itself lead to the absence of intra-saccadic visual motion experiences (Campbell & Wurtz, 1978). Being flanked by the pre- and post-saccadic image, the intra-saccadic motion transient becomes masked and does not appear in conscious perception (Castet, Jeanjean, & Masson, 2002). Presenting an analogous sequence during fixation reduces the visibility of the brief motion stimulation when it is temporally flanked by stationary images (Duyck, Wexler, Castet, & Collins, 2018). In experiments designed to test the active omission mechanism, visual contrast sensitivity has been assessed around the time of a saccade by asking participants to report the contrast polarity of a briefly flashed Gaussian stimuli (Burr, Morrone, & Ross, 1994; Knöll, Binda, Morrone, & Bremmer, 2011). Contrast sensitivity declined around ∼50 to 100 ms before saccade initiation, reached a minimum at saccade onset, and recovered shortly afterward. The time course of contrast reduction was interpreted as the signature of an active mechanism that suppresses visual input around saccade execution (Ross, Morrone, Goldberg, & Burr, 2001). It was argued that suppression reflects a transient reduction of processing in the magnocellular pathway around the time of saccades (Burr et al., 1994; Ross, Burr, & Morrone, 1996).

The idea that visual motion processing is uniformly suppressed during saccades was challenged later on. Castet and Masson (2000) demonstrated that motion perception can be preserved for stimuli optimized for intra-saccadic vision. Specifically, they showed gratings that drifted with a temporal frequency higher than the flicker fusion frequency, thus making them invisible during fixation. When observers executed a saccade across these stimuli, however, the motion became visible because successive stimulus frames were sampled by different retinal locations. The authors proposed that omission of intra-saccadic motion arises passively through backward masking by the post-saccadic input (Castet et al., 2002). To test this idea, they presented stationary gratings at various times relative to saccade onset. When the grating fell entirely within the period of saccade execution, participants reported the grating as moving. However, when its presentation extended beyond the time of saccade execution, the grating was more often perceived as stationary. When the stimulus remained visible after saccade offset, the stationary post-saccadic input may have served as a backward mask that suppressed awareness of the intra-saccadic stimulation (Castet et al., 2002). In this view, no active mechanism is needed, as saccadic omission would be handled passively by the temporal dynamics of visual processing. Recent studies even suggest that sensitivity to intra-saccadically presented stimuli is reduced as early as the retina itself (Hafed, Idrees, & Baumann, 2024; Idrees, Baumann, Franke, Münch, & Hafed, 2020; Idrees et al., 2022).

Schweitzer, Doering, Seel, Raisch, and Rolfs (2025) have suggested that the visual system has evolved to routinely disregard the sensory consequences of its own eye movements while remaining sensitive to genuine high-speed motion in the environment. In their study, participants judged the curvature of motion trajectories during fixation and found that visibility thresholds for high-speed motion shift in accordance with the main sequence, which describes the relationship between saccade amplitude and peak velocity (Rolfs, Schweitzer, Castet, Watson, & Ohl, 2025; Schweitzer et al., 2025). From this perspective, saccadic omission may not require a dedicated, efference-copy–driven “suppression module.” Instead, the limits of motion visibility may be intrinsically tuned to the kinematic constraints that govern saccade generation (Rolfs et al., 2025; Schweitzer et al., 2025).

Because the main sequence is stable within observers, the speed of the visual stimulation that will be on the retina when executing a planned saccade is predictable. Saccade-contingent predictions might lead to a selective omission of only that visual motion speed actually produced by a saccade. Consistent with this idea, we have shown that the sensorimotor system habituates and omits motion when a certain visual speed is repeatedly coupled to a certain saccade vector (Pomè, Schlichting, Fritz, & Zimmermann, 2024; Pomè & Zimmermann, 2025; Zimmermann, 2020). In those experiments, we used stimuli optimized for intra-saccadic vision and repeatedly paired specific motion speeds with specific saccade vectors, including speeds that violated the kinematic predictions derived from the main sequence. After repeated exposure to the novel motion speeds, sensitivity for these stimulations declined. Importantly, the decline was selective to the saccade vector executed during motion presentation (Pomè et al., 2024; Pomè & Zimmermann, 2025).

In the present study, we aimed to compare the effectiveness of intra-saccadic habituation and of post-saccadic masking by quantifying both effects within interleaved trial types in the same session. Masking studies usually are carried out with suddenly appearing or disappearing (i.e., flashed) stimuli (Castet et al., 2002). By contrast, stimuli optimized for intra-saccadic vision, as devised by Castet and Masson (2000), produce relatively smooth retinal stimulation. We wondered whether post-saccadic visual masking also works for stimuli with smooth rather than sudden onsets and offsets. In order to directly compare habituation and masking as contributors to saccadic omission, we quantified their effects both in separate blocks and within the same session.

## Methods

### Participants

Thirteen subjects (nine females, mean age = 26.9 years, *SD* = 6.6) participated in the study. They gave written informed consent and received compensation for their participation. Seven subjects participated in both experiments, four subjects took part only in Experiment 1 (11 subjects in total), and two more only in Experiment 2 (nine subjects in total). All observers had normal or corrected-to-normal vision (two subjects wore glasses, two contact lenses). Two participants did not complete Experiment 1 and were therefore excluded due to insufficient data (nine remaining subjects). The experiments were conducted in agreement with the Declaration of Helsinki, with the exception of pre-registration, and approved by the ethics committee of the Faculty of Mathematics and Natural Sciences of the Heinrich Heine University Düsseldorf.

### Apparatus

Stimuli were presented on a 27-inch XB272 LCD monitor (Acer, New Taipei City, Taiwan) connected to an Alienware Aurora R7 computer (Dell Technologies, Round Rock, TX), featuring a refresh rate of 240 Hz (with a frame duration of 4.16 ms) and a resolution of 1920 × 1080 pixels (with maximum and minimum luminance of 92.96 and 0.3 cd/m^2^, respectively). The monitor was calibrated to ensure accurate representation of the specified grayscale values. The experimental software was developed using MATLAB 2016b (MathWorks, Natick, MA) in conjunction with Psychtoolbox. Horizontal eye movements of the left eye were recorded using an infrared video eye tracker (EyeLink 1000; SR Research, Ottawa, ON, Canada) at a sampling frequency of 1000 Hz. The system identified start and end of saccades when eye velocity exceeded or fell below 30°/s. Participants were positioned with a chin and head rest. A standard five-point calibration was performed prior to each phase and condition.

### Stimuli

Two red crosses (0.564° × 0.564°; fixation and target) were presented at a distance of ±15° from the center of the screen to guide the performance of a 30*°* saccade. Intra-saccadic visual stimuli were full-screen-width sinusoidal gratings (spatial frequency = 0.1333, 0.1231 and 0.1143 cycles per degree of visual angle [c/°], randomized across trials), presented with a Michelson contrast (intensity difference between the lightest and darkest portions of the patch) of 10%, either stationary or drifting in rightward direction (all temporal frequency = 80 Hz, resulting in 600, 650, and 700°/s).

### Procedure: Experiment 1

Each trial started with the presentation of a fixation cross presented 15° to the left of screen center. After 1000 ms, the fixation cross was replaced by a target cross positioned 15° to the right of screen center. As soon as the target cross appeared, participants were required to direct their gaze from the fixation to the target cross. After they had completed their saccade, participants reported whether they had perceived motion during execution of the saccade by pressing the indicated keys of the keyboard. In each condition, one of three different motion stimuli was displayed on a uniformly gray background.

**Figure 1.**
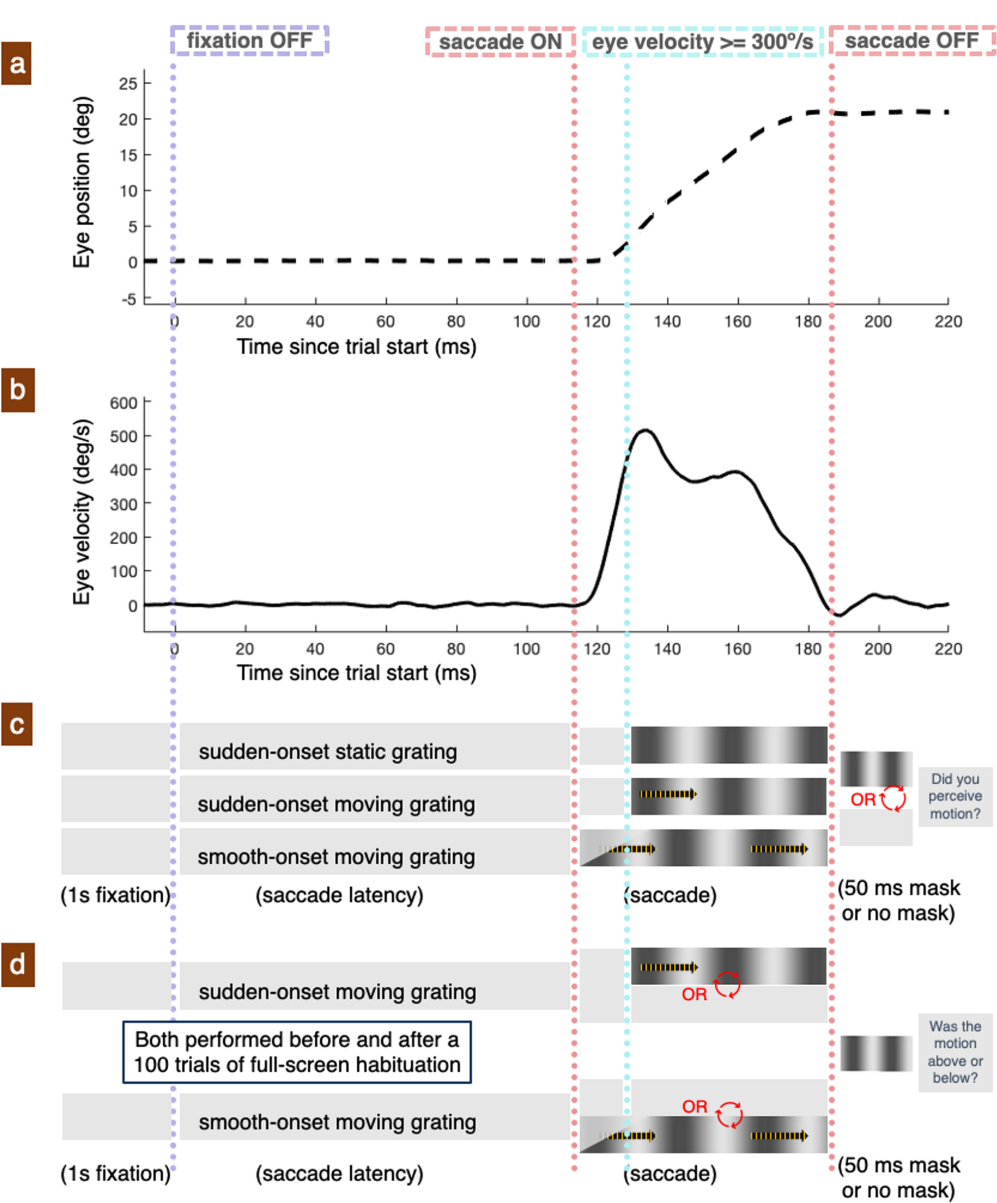
(**a**) Horizontal eye position over time. At time 0 ms, the left fixation cross disappears and the right cross appears, prompting a saccade. After 1000 ms of fixation, the eye leaves the left cross (saccade onset) and its position increases until it reaches the right cross (saccade offset). (**b**) Eye velocity over time. Velocity is zero during fixation, rises at saccade onset, exceeds the 300°/s threshold used for stimulus triggering, reaches a peak, and then decreases to near zero at saccade offset. A smaller second peak reflects a corrective saccade. (**c**) Conditions of Experiment 1. In the sudden-onset stationary and sudden-onset moving conditions, the grating appeared when eye velocity exceeded 300°/s (cyan line) and disappeared when velocity dropped below 30°/s. In the smooth-onset moving condition, a high-speed grating was present throughout the trial and became visible when the eye started moving; saccade offset triggered the 60-ms mask in half of the trials and a gray screen in the other half, followed by a black full-screen flash. Afterward, the newly gray screen featured the task question to the subject. (**d**) Conditions of Experiment 2. Two half-screen motion conditions were tested in two phases, before and after 100 habituation trials. In the sudden-onset condition, motion was triggered when eye velocity exceeded 300°/s. In the smooth-onset condition, motion became visible with saccade onset. In both conditions, saccade offset triggered a full-screen mask.

Participants underwent three conditions of 70 trials each. The first condition motion stimulus was a sudden-onset stationary sinusoidal grating, covering the whole screen (Figure 1C, sudden-onset static grating). Presentation of the grating was triggered when eye velocity exceeded 300°/s and was removed when velocity fell below 30°/s. A previous study showed that this manipulation yields a strong impression of motion due to the saccade-induced retinal image translation (Castet et al., 2002). The second condition motion stimulus involved the same stimulus during the saccade but drifted rightward on the screen (Figure 1C, sudden-onset moving grating). The third condition motion stimulus was a rightward-drifting high-speed grating that was present already during the initial fixation and remained on the screen until 60 ms after saccade offset (Figure 1C, smooth-onset moving grating). Because the drift rate was above the critical flicker fusion frequency, the stimulus appeared uniform during ocular fixation. However, it became visible during the saccade because retinal sampling across eye movement effectively slowed the pattern on the retina, producing a smooth perceptual onset (Castet & Masson, 2000). In all three conditions, the stimulus was removed when velocity fell below 30°/s (sudden-onset condition), or later simultaneously with the appearance of the black full-screen flash (smooth-onset condition). The resulting motion percept had a smooth offset. The post-saccadic interval (1000 ms) contained either a uniform gray screen (no-mask trials) or a mask appearing immediately after the intra-saccadic stimulus (at the saccadic offset) and consisting of a stationary grating flashed for 60 ms followed by a gray background. A 50-ms black full-screen flash, presented in all conditions, was shown to mask the offset transient of the drifting grating present in the post-saccadic interval of the no-mask trials in the third condition. The first 10 trials of each condition were treated as training, and they were not included in the analyses.

### Procedure: Experiment 2

Participants underwent two conditions (sudden-onset motion and smooth-onset motion), each composed of two phases (pre- and post-habituation). In both conditions (Figure 1D), the moving grating was presented in either the upper or the lower half of the screen, in 50 pre-habituation trials. On every trial, a post-saccadic mask was presented for 60 ms at saccade offset, and participants reported whether the target had appeared in the upper or lower half (two-alternative forced choice [2AFC]). The manipulation provided a direct comparison of masking effects for the two motion stimuli (partial replication of Experiment 1). The post-habituation trials were preceded by habituation trials. During the habituation trials, participants completed 100 trials with a full-screen grating presented according to the stimulus dynamics of the respective condition, ensuring repeated exposure. Subjects performed saccades, but no perceptual task was required. In the subsequent post-habituation trials, the same 2AFC task as in pre-habituation was presented for 50 trials. To maintain the habituation state throughout testing, blocks of five test trials were interleaved with five additional habituation trials (full-screen; no response) (Pomè et al., 2024). The order of upper/lower trials was always randomized, whereas phase order was held constant (pre-habituation, habituation, post-habituation) within each motion stimulus condition.

### Data analyses

Eye tracking data were used to exclude invalid trials from the behavioral data. Specifically, a trial was considered invalid when the saccade started too early (i.e., before fixation cross offset), started or finished too late (1000 ms after the fixation offset), when its amplitude did not exceed 50% of the required distance (=15°), or when participants blinked during saccade execution. Data from Experiment 1 were analyzed with a 2 × 3 repeated-measures analysis of variance (ANOVA). The two factors were the motion stimulus (sudden-onset retinal motion, sudden-onset screen motion, and smooth-onset screen motion) and the trial type (masked motion vs. not masked motion trials). For data from Experiment 2, a 2 × 2 repeated-measures ANOVA was performed, with the motion stimulus (sudden-onset screen motion and smooth-onset screen motion) as the first factor and the phase compared with habituation (pre- vs. post-habituation) as the second factor. In order to compare the saccade metrics of Experiment 2 across pre-habituation and post-habituation phases, six paired-sample *t*-tests were performed for peak velocities, durations, and amplitudes of both motion conditions.

## Results

### Experiment 1

**Figure 2.**
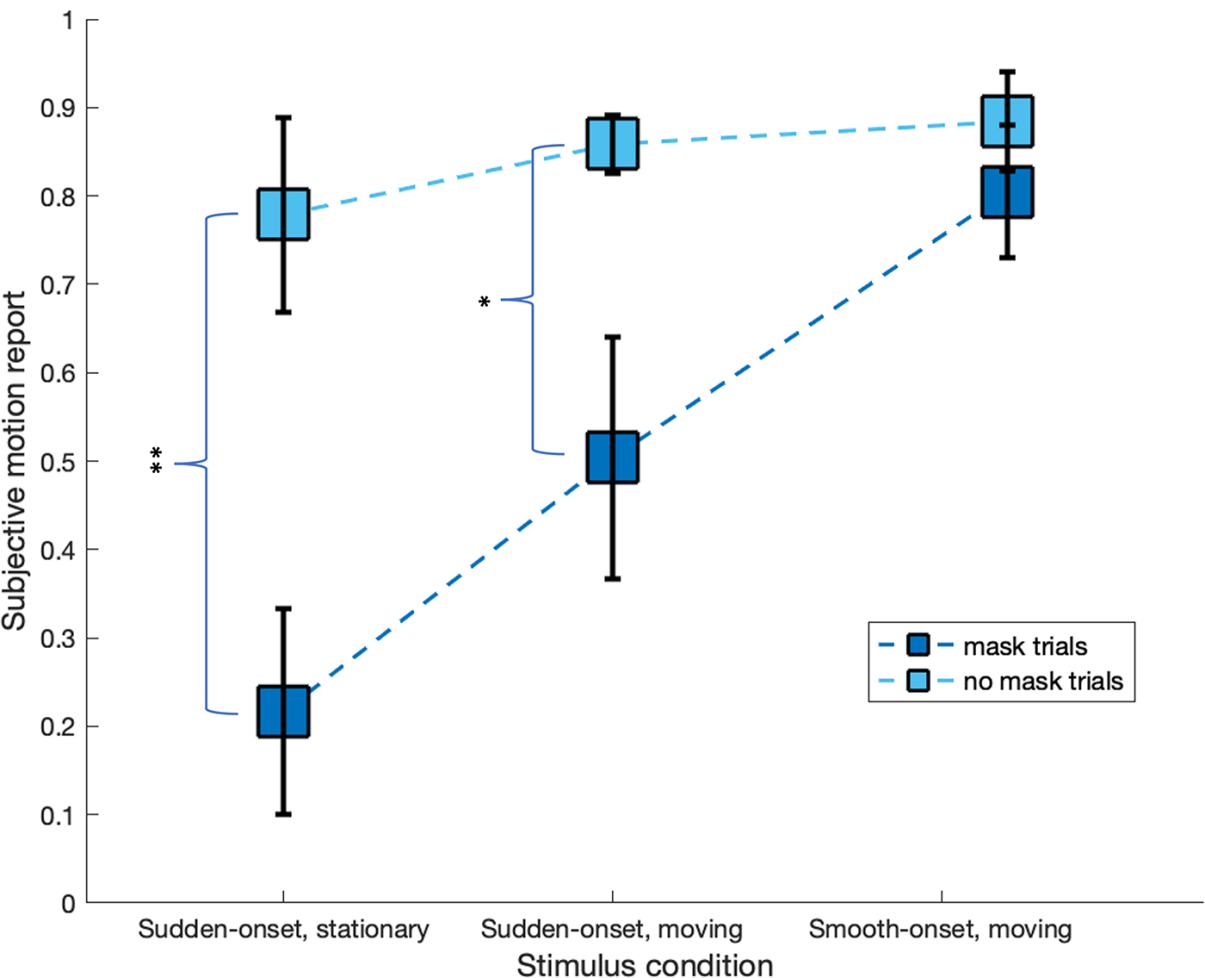
Results of the three conditions of Experiment 1 as indicated by the *x*-axis ticks. These are divided by trial type to show the differences between the detection rates of the post-saccadically masked motion (dark blue) and the not-masked one (light blue). The results show the average rate of subjective motion report across participants. The asterisks show the significant differences: masked trials versus not-masked trials of sudden-onset stationary condition (**p < 0.01) and masked trials versus not-masked trials of sudden-onset moving condition (*p < 0.05). Error bars represent standard error of the mean (*SEM*) across participants.

In Experiment 1, post-saccadic visual masking and intra-saccadic habituation were tested with three intra-saccadic stimuli: sudden-onset stationary gratings (replicating Castet et al., 2002), sudden-onset moving gratings, and smooth-onset moving gratings. Visual subjective motion report rates (proportion of trials reporting motion) are shown in Figure 2.

In the sudden-onset stationary condition, participants reported visual motion on 77.8% ± 11.0% of unmasked trials but only 21.6% ± 11.6% of masked trials. For sudden-onset moving gratings, detection was 85.8% ± 3.3% (unmasked) versus 50.4% ± 13.6% (masked). Smooth-onset moving gratings yielded high detection regardless of masking: 88.4% ± 5.6% (unmasked) versus 80.5% ± 7.5% (masked). A 2 (mask: present/absent) × 3 (motion stimulus) repeated-measures ANOVA revealed a significant main effect of mask, *F*(1, 8) = 29.7, *p* < 0.001, $\eta_{p}^{2}$ = 0.79; a main effect of motion stimulus, *F*(2, 16) = 4.8, *p* = 0.023, $\eta_{p}^{2}$ = 0.37; and a marginal interaction, *F*(2, 16) = 3.2, *p* = 0.066, $\eta_{p}^{2}$ = 0.29.

Post hoc paired *t*-tests (Bonferroni-corrected) confirmed significant post-saccadic masking for sudden-onset stationary, difference = 56.2, *t*(8) = 4.2, *p* = 0.003, *d* = 1.97; and sudden-onset moving, difference = 35.5, *t*(8) = 2.6, *p* = 0.033, *d* = 1.2; but not smooth-onset moving, difference = 7.9, *t*(8) = 0.8, *p* = 0.463, *d* = 0.3. These results suggest that post-saccadic masking depended on the stimulus: It was strongest for sudden-onset stationary gratings, weaker for sudden-onset moving gratings, and minimal for smooth-onset moving gratings.

### Experiment 2

**Figure 3.**
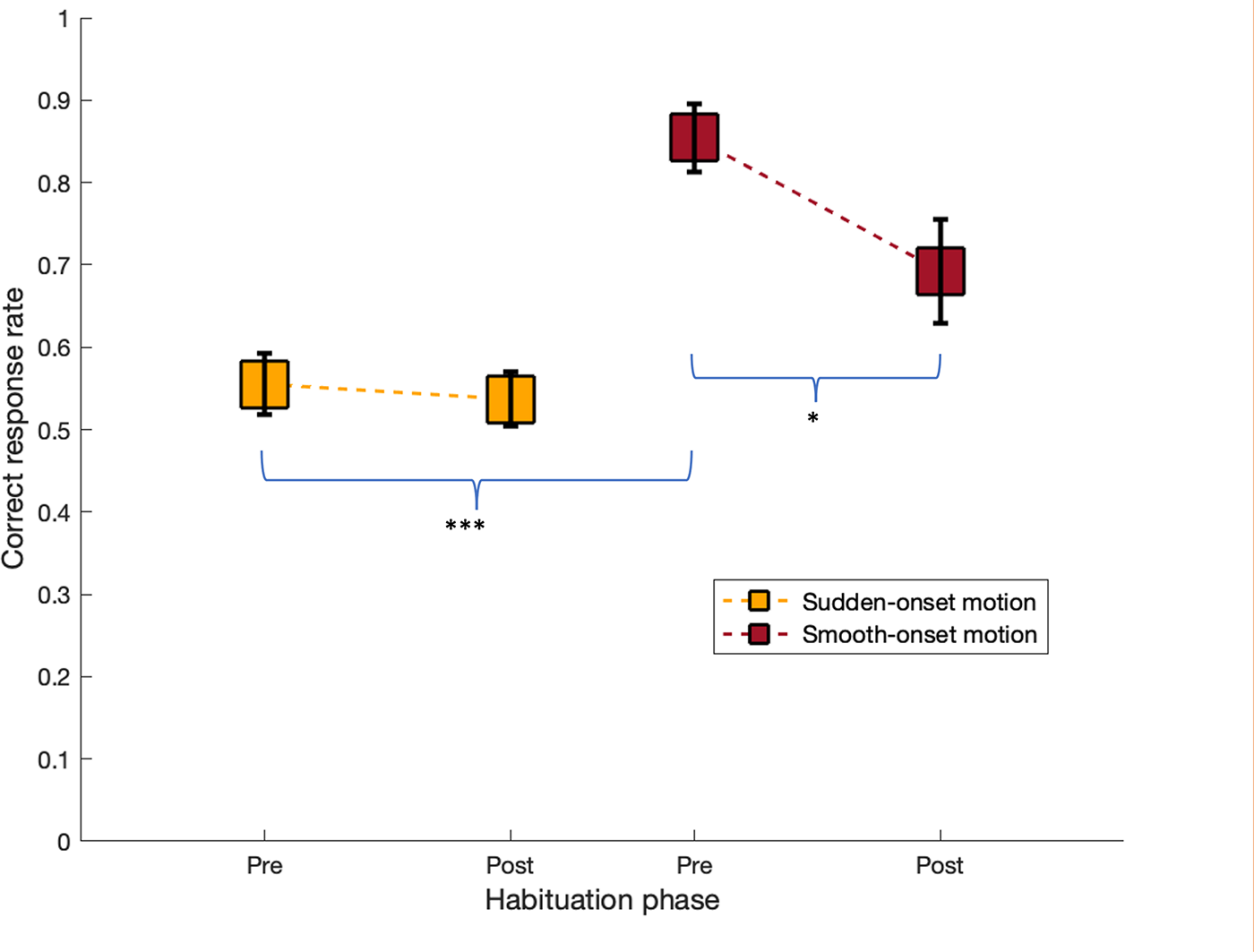
Results of the two conditions of Experiment 2 (sudden-onset in orange, smooth-onset in red) showing the average correct response rate across participants. The *x*-axis shows an additional division by phase to show the differences between pre-habituation (first and third on the *x*-axis from the right) and post-habituation (second and fourth). The asterisks show the significant differences: sudden-onset pre-habituation versus smooth-onset pre-habituation (***p < 0.001) and smooth-onset pre-habituation versus smooth-onset post-habituation (*p < 0.05). Error bars represent *SEM* across participants.

Experiment 2 compared visual motion detection (proportion correct in 2AFC location task: upper/lower half of the screen) for sudden-onset versus smooth-onset moving gratings before and after habituation. Results are shown in Figure 3, which plots the mean proportion correct ± *SEM* across each phase. Before the habituation, performance was near chance for sudden-onset (55.5% ± 3.7%) but high for smooth-onset (85.4% ± 4.1%). After the habituation, sudden-onset remained low (53.7% ± 3.3%), but smooth-onset dropped to 69.2% ± 6.3%. A 2 (motion stimulus: sudden/smooth) × 2 (phase: pre-habituation/post-habituation) repeated-measures ANOVA showed a main effect of motion stimulus, *F*(1, 8) = 23.83, *p* = 0.001, $\eta_{p}^{2}$ = 0.75; a main effect of phase approaching significance, *F*(1, 8) = 5.01, *p* = 0.056, $\eta_{p}^{2}$ = 0.39; and no interaction, *F*(1,8) = 4.18, *p* = 0.075, $\eta_{p}^{2}$ = 0.34.

Post hoc tests (Bonferroni-corrected) confirmed higher pre-habituation performance for smooth-onset versus sudden-onset, difference = 29.9, *t*(8) = 5.39, *p* < 0.001, *d* = 2.21; and a decrease after habituation for smooth-onset stimuli, difference = 16.2, *t*(8) = 2.74, *p* = 0.026, *d* = 1.197; but not sudden-onset stimuli, difference = 1.8, *t*(8) = 0.386, *p* = 0.709, *d* = 0.13. Thus, intra-saccadic habituation reduced sensitivity to smooth-onset motion but did not affect sudden-onset motion.

**Figure 4.**
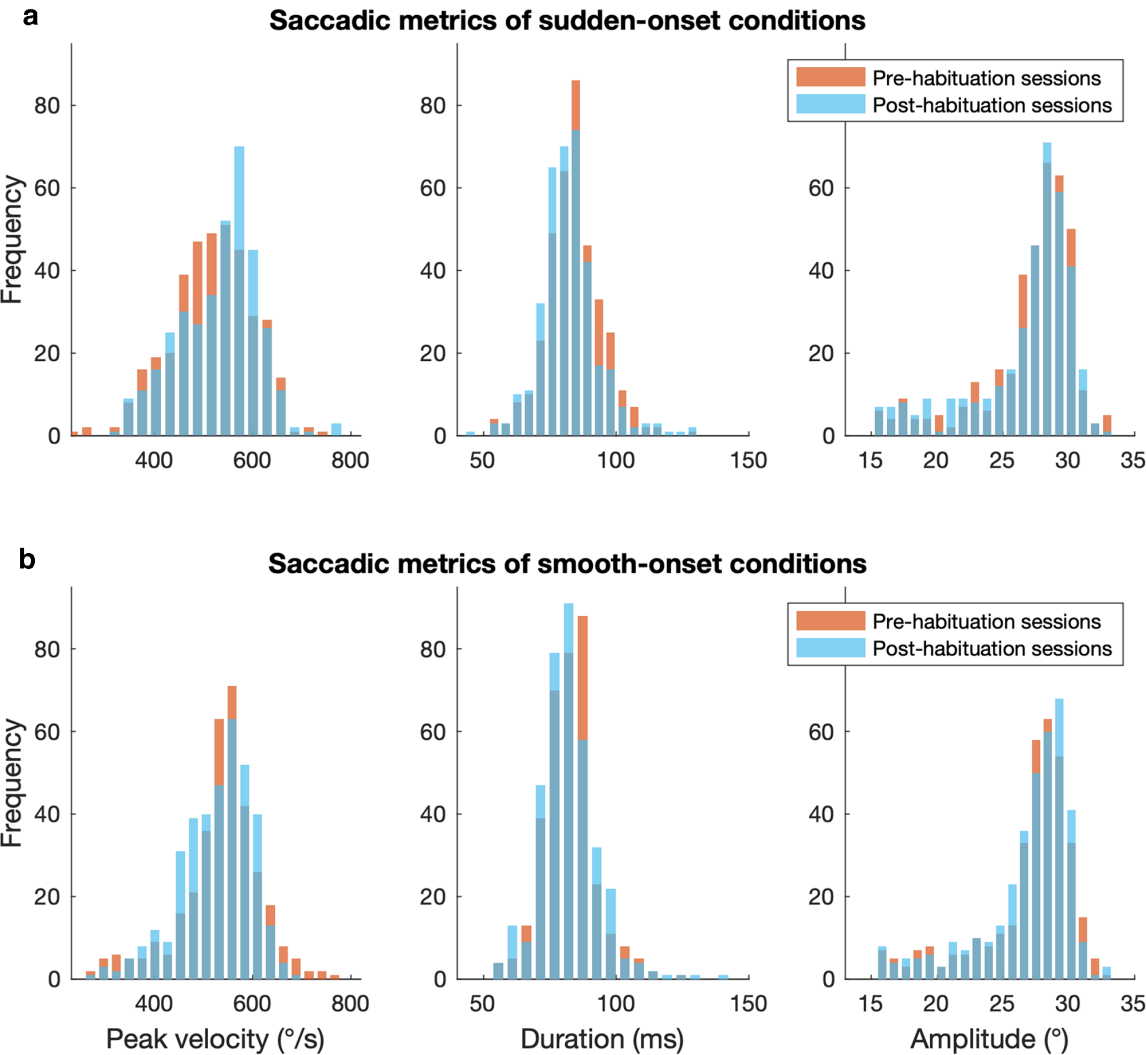
Saccade metrics of pre-habituation (orange) and post-habituation (blue) phases for (**a**) sudden-onset and (**b**) smooth-onset motion conditions. Frequency histograms of peak velocity (°/s), duration (ms), and amplitude (°) are shown for each phase. The degree of overlap of the pre-habituation and post-habituation distributions across all three metrics demonstrates that saccade kinematics remained stable over time, indicating that the habituation procedure did not produce systematic declines attributable to oculomotor fatigue.

Figure 4 compares the saccade metrics of the pre-habituation phase with the corresponding post-habituation phase of the sudden-onset motion conditions (top row) and of the smooth-onset motion conditions (bottom row). In both the sudden-onset and the smooth-onset conditions, the frequencies of peak velocity, duration, and amplitude are almost entirely superimposed for pre-habituation and post-habituation trials, with no systematic shift toward lower velocities, longer durations, or smaller amplitudes. Because fatigue is reflected in reduced saccade peak velocities, shortened saccade amplitudes, and prolonged durations across time-on-task paradigms (Chen, Kuo, Hsu, & Wang, 2022), the preserved alignment of these distributions indicates that the habituation phase did not induce measurable oculomotor fatigue. To assess potential oculomotor fatigue quantitatively, we compared saccade metrics before habituation and after habituation with paired-samples *t*-tests for each condition. Between the smooth-onset pre-habituation and post-habituation phases, there were no difference for peak velocity, *t*(19) = 0.574, *p* = 0.573; duration showed no reliable change, *t*(19) = 0.535, *p* = 0.599; and amplitude was likewise stable, *t*(19) = 0.911, *p* = 0.374. No differences were found between the sudden-onset pre-habituation and post-habituation phases: peak velocity, *t*(19) = –0.262, *p* = 0.796; duration, *t*(19) = –0.354, *p* = 0.727; amplitude, *t*(19) = –0.488, *p* = 0.631). Thus, any changes observed in the main experimental measures are unlikely to be explained by a fatigue-related degradation of basic saccade metrics but rather reflect specific effects of the visual motion conditions or habituation procedure.

## Discussion

The present study aimed to disentangle the relative contributions of post-saccadic masking and intra-saccadic habituation to saccadic omission. In two experiments, we compared how sudden-onset versus smooth-onset motion stimuli are perceived during the execution of saccades. Our findings demonstrate that the post-saccadic masking and intra-saccadic habituation operate differently and may contribute to visual stability through distinct temporal and sensory pathways.

In Experiment 1, visual subjective motion report strongly depended on both the onset characteristics of the stimulus (sudden or smooth) and the presence of a post-saccadic mask. Consistent with Castet et al. (2002), sudden-onset gratings that appeared during the saccade and that were followed by a static post-saccadic image were rarely perceived as moving. This result supports the idea that visual masking effectively prevents the perception of intra-saccadic motion when intra-saccadic stimulation has a sudden onset. However, post-saccadic masking was weakened when the stimulus drifted. Furthermore, masking was largely ineffective for smooth-onset stimuli that were optimized for intra-saccade vision.

Experiment 2 further revealed that repeated exposure to smooth-onset intra-saccadic visual motion leads to a decline in sensitivity (i.e., intra-saccadic habituation). Although sudden-onset moving gratings were barely detected before and after habituation, smooth-onset motion, which was initially well detected, showed a significant reduction in discrimination performance after repeated exposure. The decline suggests that intra-saccadic habituation acts as an adaptive, experience-dependent process that reduces sensitivity to predictable motion patterns associated with eye movements. Together, these findings indicate that post-saccadic masking primarily affects stimuli that are presented with sudden onsets during saccade execution. In natural vision, however, objects do not suddenly appear while the eyes are in flight. The close correspondence between post-saccadic masking and visual masking phenomena observed during fixation therefore appears to depend critically on the use of stimuli with sudden temporal onsets.

Two aspects of our experimental design lack ecological validity. First, the stimulus, which was optimized for intra-saccadic viewing, drifted at a high speed. Under natural viewing conditions, objects do not typically move at such velocities during saccades. Importantly, however, the retinal motion induced by our stimulus is comparable to the retinal image motion generated when a stationary scene is swept across the retina during a saccade. Specifically, we presented gratings drifting rightward at a speed of ∼650°/s while participants executed rightward saccades, resulting in a net retinal motion ranging approximately between 150°/s to 300°/s. A saccade of 5*°* across a static visual scene would produce a comparable retinal speed.

Second, although the intra-saccadic stimulus had a smooth temporal onset, the post-saccadic mask was presented with a sudden onset. In natural vision, both the intra-saccadic stimulation and the transition to the post-saccadic image are temporally smooth, reflecting the monotonic increase and subsequent decrease in saccade velocity. Consequently, the suddenly presented post-saccadic stimulus in our experiment may not constitute an optimal mask for an intra-saccadic stimulus with a smooth onset. Generating a post-saccadic stimulus with a similarly smooth temporal profile, however, is technically very challenging. An alternative approach to generating a temporally smooth post-saccadic stimulus could involve presenting a stationary mask already before saccade offset. A primary concern is that the stationary mask and the intra-saccadic moving stimulus could not be presented at the same spatial location. Two technical solutions seem possible: (a) the moving intra-saccadic stimulus could stop shortly before saccade end and be replaced by the mask, or (b) the mask could be shown in the peripheral field of view while the moving stimulus remains in the center. We anticipate that both may present methodological challenges for observers to accurately report what was perceived intra-saccadically. This approach warrants investigation in future studies to determine its feasibility and impact on perceptual reports.

Our results suggest that stimuli with sudden versus smooth temporal onsets lead to qualitatively different conclusions regarding saccadic omission. Perception of sudden-onset stimuli is profoundly altered when they are presented around the time of saccades: Such stimuli appear reduced in contrast (saccadic suppression), displaced toward the saccade target (saccadic compression of space), and temporally mislocalized (saccadic compression of time). In contrast, stimuli designed for intra-saccadic viewing do not appear reduced in intensity (García-Pérez & Peli, 2011), nor is their motion omitted (Castet & Masson, 2000).

Notably, previous studies investigating both the saccadic suppression hypothesis (Burr et al., 1994) and the saccadic masking hypothesis (Castet et al., 2002) have predominantly relied on stimuli with sudden onsets. For intra-saccadic motion with a smooth onset, our results indicate that backward masking alone cannot account for saccadic omission and that habituation provides an experience-dependent mechanism to stabilizing perception across saccades.
